# Controlled afterglow luminescent particles for photochemical tissue bonding

**DOI:** 10.1038/s41377-022-01011-3

**Published:** 2022-10-27

**Authors:** Seong-Jong Kim, Minji Choi, Guosong Hong, Sei Kwang Hahn

**Affiliations:** 1 Department of Materials Science and Engineering, Pohang University of Science and Technology (POSTECH), 77 Cheongam-ro, Nam-gu, Pohang, Gyeongbuk 37673 Korea; 2 Department of Materials Science and Engineering, Stanford University, Stanford, CA 94305 USA

**Keywords:** Biophotonics, Biomedical materials, Tissues

## Abstract

Upconversion materials (UCMs) have been developed to convert tissue-penetrating near-infrared (NIR) light into visible light. However, the low energy conversion efficiency of UCMs has limited their further biophotonic applications. Here, we developed controlled afterglow luminescent particles (ALPs) of ZnS:Ag,Co with strong and persistent green luminescence for photochemical tissue bonding (PTB). The co-doping of Ag^+^ and Co^2+^ ions into ZnS:Ag,Co particles with the proper vacancy formation of host ions resulted in high luminescence intensity and long-term afterglow. In addition, the ALPs of ZnS:Ag,Co could be recharged rapidly under short ultraviolet (UV) irradiation, which effectively activated rose bengal (RB) in hyaluronate-RB (HA-RB) conjugates for the crosslinking of dissected collagen layers without additional light irradiation. The remarkable PTB of ZnS:Ag,Co particles with HA-RB conjugates was confirmed by in vitro collagen fibrillogenesis assay, in vivo animal wound closure rate analysis, and in vivo tensile strength evaluation of incised skin tissues. Taken together, we could confirm the feasibility of controlled ALPs for various biophotonic applications.

## Abstract (web summary)

Afterglow luminescent particles of ZnS:Ag,Co are developed as an efficient light delivery agent for photochemical tissue bonding in deep tissues with prolonged luminescence.

## Graphical abstract

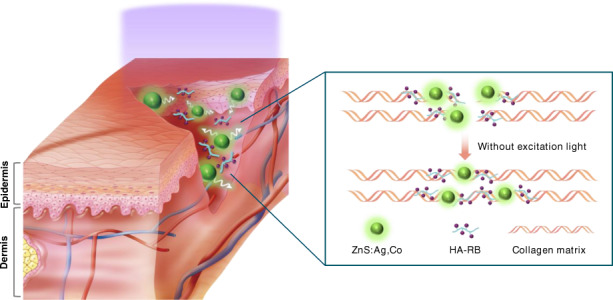

## Introduction

A variety of wound closures, including bioadhesives, have been developed as an alternative to traditional sutures^1,2^. Nevertheless, the clinical application of wound closures has still been limited due to the low tissue bonding strength^3,4^. Recently, photochemical tissue bonding (PTB) has emerged as a novel technique for wound closing. The PTB has a wound healing efficiency comparable to that of sutures with the advantage of overcoming several problems of sutures, such as secondary inflammation and needle puncturing^5,6^. This technique uses light and photosensitizers to promote collagen crosslinking and reduce inflammation and scarring^7^. Rose Bengal (RB) dye is one of the most common photosensitizers for PTB^8^. RB absorbs energy from green light and interacts with collagen to produce collagen free radicals. These radical species initiate the formation of covalent bonding between collagen molecules. When the incision is closed, the light transmission efficiency decreases, because the green light is attenuated depending on the tissue penetration depth^9^. Previously, upconversion nanoparticles (UCNPs) were applied to effectively activate RB in the deep tissue and induce collagen crosslinking for PTB^10^. UCNPs converted the tissue-penetrating NIR light into a green light to deliver the light noninvasively into the deep tissue. However, the low quantum yield of UCNPs necessitated multiple and long-term treatments for PTB^11,12^.

In this regard, afterglow luminescent particles (ALPs) can be effectively harnessed for PTB. ALPs are materials with optical properties that trap the irradiated photoenergy in defect states and slowly emit the stored energy as light. Several kinds of materials have been used to synthesize ALPs, including oxides^13^, non-oxides^14^, and organic materials^15^. It has been reported that ALPs can sustain emission for seconds to days after ceasing light irradiation^16–18^. These unique properties of ALPs can overcome the limited penetration depth of the external light and prevent damage to normal tissues caused by repeated and prolonged light irradiation. Furthermore, ALPs can be tuned to have high luminescence efficiency in the visible light spectrum^19,20^, which would improve the efficiency of conventional PTB. To our best knowledge, however, there has been no report on the application of ALPs for PTB.

**Fig. 1:**
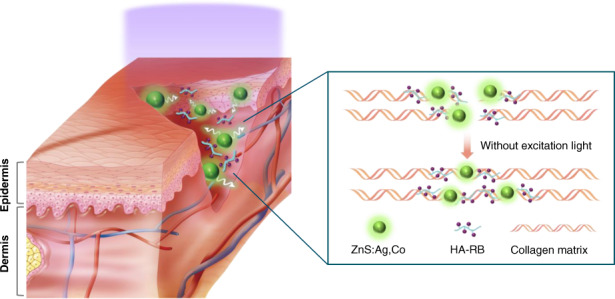
Schematic illustration. The photochemical tissue bonding of incised collagen matrix by HA-RB/ZnS:Ag,Co mixtures under or after UV light irradiation

Here, we have developed ALPs of ZnS:Ag,Co as a light delivery agent for PTB in the deep tissue with prolonged luminescence. As shown in Fig. 1, ZnS:Ag,Co particles can transmit high-intensity green luminescence to hyaluronate-RB (HA-RB) conjugates for continuous and prolonged PTB after short-time ultraviolet (UV) irradiation. HA has been widely used as a transdermal delivery carrier for chemical and protein drugs due to its property that promotes penetration into the skin barrier^21,22^. Accordingly, HA-RB conjugates can be widely distributed in the collagen layer and effectively activate collagen crosslinking with green light irradiation^10^. After in vitro characterization and luminescence analysis of ZnS:Ag,Co particles, we assessed and discussed the effect of mixtures of ZnS:Ag,Co particles with HA-RB conjugates (HA-RB/ZnS:Ag,Co mixture) on the PTB in BALB/c mice.

## Results

### Preparation and characterization of ZnS:Ag,Co particles and HA-RB conjugates

**Fig. 2:**
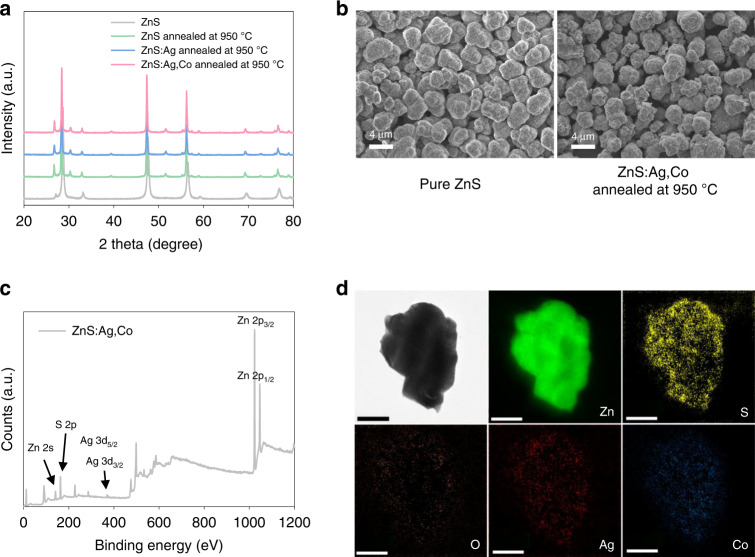
Characterization of ZnS:Ag,Co particles. **a** XRD spectrum of ZnS, ZnS:Ag, and ZnS:Ag,Co particles with or without annealing at 950 °C under argon atmosphere. **b** SEM images of pure ZnS and ZnS:Ag,Co particles annealed at 950 °C. **c** XPS spectrum and **d** EELS elemental mapping images of ZnS:Ag,Co particles after calcination, respectively (scale bar = 500 nm)

The ALPs of ZnS:Ag,Co were synthesized by the solid-state reaction at 950 °C under an argon atmosphere for 3 h, which was determined by the optimization process in terms of afterglow luminescence (AL) (Supporting Information, Figs. S1 and S2). X-ray diffraction (XRD) revealed the phase transition during annealing. The peak of XRD showed the dominant wurtzite structure in ZnS:Ag,Co particles, and no other diffraction peaks were observed in the dopant (Fig. 2a). The morphology and size of ZnS:Ag,Co particles were characterized by scanning electron microscopy (SEM). The calculated size of the ZnS:Ag,Co particle was 2.91 ± 0.343 μm, a slight increase compared to the pure ZnS particle (Fig. 2b). In Fig. 2c, the presence of Ag^+^ and Co^2+^ dopant was confirmed by X-ray photoelectron spectroscopy (XPS) and high-resolution transmission electron microscopy (HRTEM) with electron energy loss spectroscopy (EELS). The binding energy peaks obtained from the XPS spectrum were 364.6 and 370.5 eV, corresponding to 3d_5/2_ and 3d_3/2_ of the Ag^+^ dopant, respectively (Supporting Information, Fig. S3). Elemental mapping using EELS confirmed the homogenous distribution of Ag^+^ and Co^2+^ dopants in ZnS:Ag,Co particles (Fig. 2d). A low oxygen level contributed to the formation of sulfur vacancies without conversion to the ZnO structure^23^.

On the other hand, the successful conjugation of RB to HA-diaminohexane (HA-DAH) was confirmed by ^1^H NMR analysis with an absorbance peak shift (Supporting Information, Fig. S4). The peaks of the HA-RB conjugate appeared at 1.38, 1.55, and 1.68 ppm, indicating methylene protons in the ^1^H NMR spectrum. The maximum absorbance of RB was measured at the wavelength of 550 nm, and the red shift was observed in the corresponding peak of the HA-RB conjugate.

### Luminescence properties and mechanism of ZnS:Ag,Co particles

**Fig. 3:**
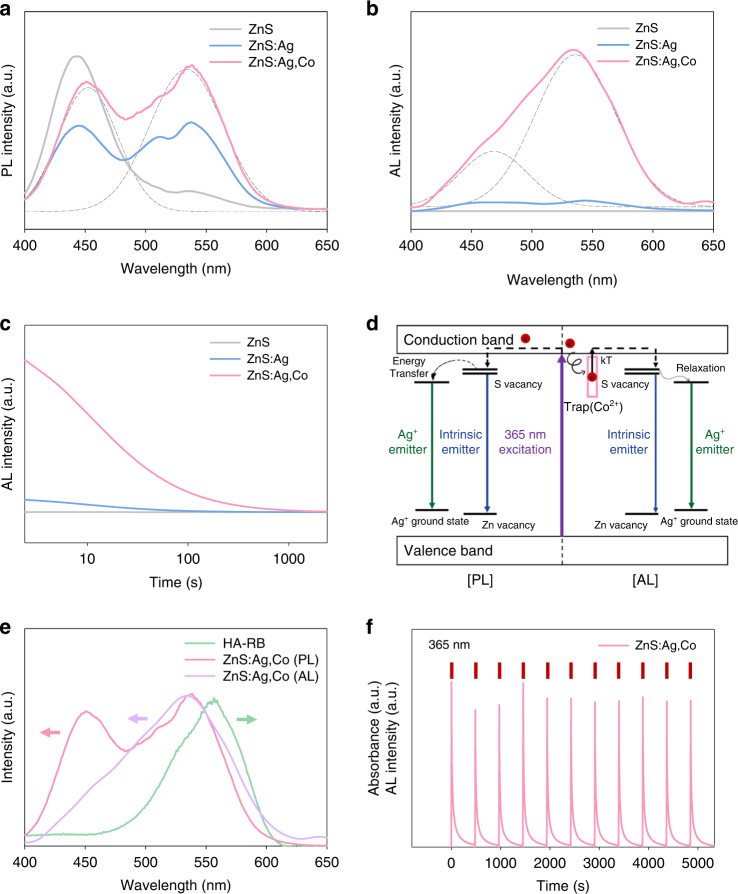
The luminescence characteristics of ZnS:Ag,Co particles. **a** PL spectrum, **b** AL spectrum, and **c** afterglow decay curves of ZnS, ZnS:Ag, and ZnS:Ag,Co particles after UV light irradiation, respectively. **d** The luminescence mechanism of ZnS:Ag,Co particles. **e** The luminescence of ZnS:Ag,Co particles and the absorbance of HA-RB conjugates. **f** Time-resolved afterglow measurements of ZnS:Ag,Co particles under repetitive 365 nm recharging light

Figure 3a shows the photoluminescence (PL) spectrum of ZnS:Ag,Co particles. When ZnS:Ag,Co particles were excited at 365 nm, two major emission bands were observed at 450 and 540 nm. The PL intensity of ZnS:Ag,Co particles was significantly increased by adding Ag^+^ and Co^2+^ dopants in the green spectral region. In addition, the AL intensity of ZnS:Ag,Co particles was 15.1 times higher than that of ZnS:Ag particles at 540 nm wavelength (Fig. 3b). There was no peak shift in the AL wavelength of ZnS:Ag,Co particles with increasing time after excitation (Supporting Information, Fig. S5). The afterglow decay of ZnS, ZnS:Ag, and ZnS:Ag,Co particles was monitored after pre-irradiation with UV light for 30 s (Fig. 3c). The afterglow of ZnS:Ag,Co particles persisted for more than 1000 s and the half-life of the afterglow was about 30 s, which was 14.4 times longer than that of ZnS:Ag particles. These results confirmed that the Co^2+^ dopant contributed as a key factor of AL by substituting the zinc site and increasing the energy trap density. As shown in Fig. 3d, the luminescence mechanism of ZnS:Ag,Co particles can be explained with a band diagram. The light absorption of the ZnS host at 365 nm light irradiation promotes electron excitation from the valence band to the conduction band. The PL peak at 450 nm originated from the intrinsic emitter between sulfur vacancy states and zinc vacancy states, whereas the peak at 540 nm resulted from the energy transfer between intrinsic emitters and Ag^+^ emitters^19,24^. Both PL peaks were prominently observed during the excitation at 365 nm. In terms of AL, the defect states created by Co^2+^ dopants act as energy traps that store excited electrons without emission^17^. At room temperature, trapped energy is slowly released by thermal perturbation and is transferred to sulfur vacancy states. Then, electrons are relaxed to lower Ag^+^ excited states, and the final recombination at the Ag^+^ luminescent center produces the AL peak at 540 nm.

The activation of HA-RB conjugates by using ZnS:Ag,Co particles was assessed via UV/vis spectrophotometry and spectrofluorometer. The absorption wavelength of HA-RB conjugates overlapped with both the PL and AL wavelength of ZnS:Ag,Co particles (Fig. 3e). It means that HA-RB conjugates can absorb the luminescence of ZnS:Ag,Co particles with or without UV light irradiation and can be activated for collagen crosslinking. The photostability and rechargeability of ZnS:Ag,Co particles were analyzed by monitoring the AL intensity after 5 s pre-irradiation with UV light every 5 min (Fig. 3f). The AL intensity peaks between repeated measurements showed less than 5% deviation, confirming the optical stability and consistency of ZnS:Ag,Co particles. These results suggested that green light converted by ZnS:Ag,Co particles could effectively and continuously activate HA-RB conjugates under or after UV irradiation.

### Light propagation of ZnS:Ag,Co particles in the incised porcine skin

**Fig. 4:**
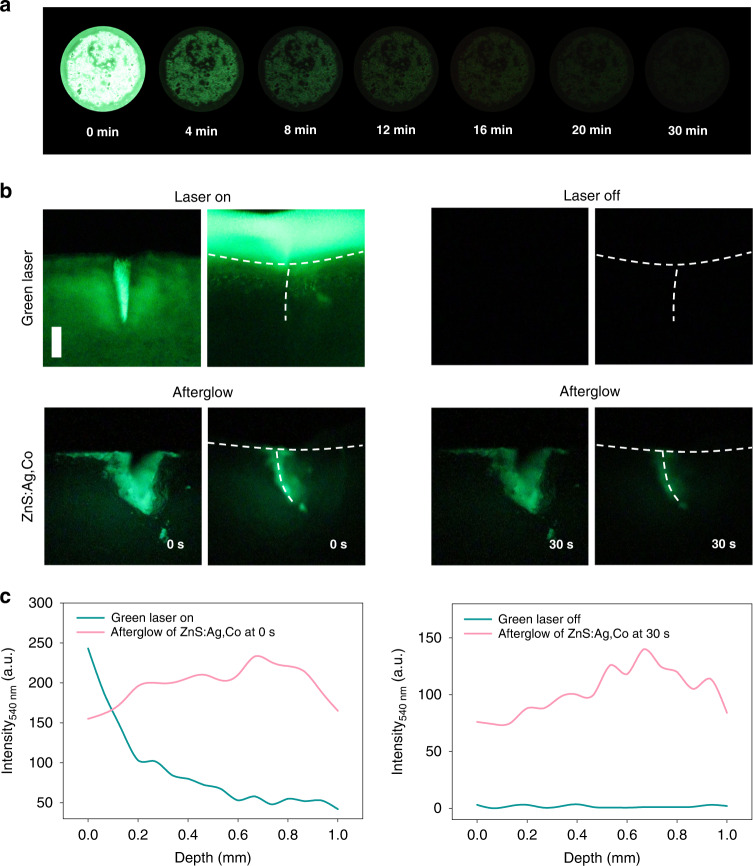
Green afterglow image of ZnS:Ag,Co particles. **a** The afterglow image of ZnS:Ag,Co particles obtained by the charge-coupled device (CCD) camera at 4, 8, 12, 16, 20, and 30 min after irradiation with a 365 nm UV lamp for 30 s. **b** The propagation of green light (upper) and light converted by ZnS:Ag,Co particles (lower) in the incision of porcine skin (scale bar = 1 mm). After irradiating the open wound with a light source, the incision site was closed and fluorescent images were taken when the green laser was turned (left) on or (right) off. Afterglow images of ZnS:Ag,Co particles were taken at 0 s and 30 s after UV light irradiation. Dashed lines indicate the incised surface of the porcine skin. **c** The green fluorescence profile in the skin depth direction of porcine tissue treated with a green light or AL of ZnS:Ag,Co particles (left: laser-on green light and the AL of ZnS:Ag,Co particles at 0 s; right: laser-off green light and the AL of ZnS:Ag,Co particles at 30 s)

The afterglow image of ZnS:Ag,Co particles shows a green light visible to the naked eye and sufficient AL (Fig. 4a). The light delivery efficiency of ZnS:Ag,Co particles into the incision was assessed using porcine skins. As shown in Fig. 4b, the green laser and the green light from ZnS:Ag,Co particles could reach into the collagen layer of the open wound. However, only ZnS:Ag,Co particles transmitted green light to the collagen layer due to the afterglow after closing the wound. The AL of ZnS:Ag,Co particles effectively remained in the deep tissue for 30 s. The scattering of the green laser appeared in the stratum corneum (SC) layer of the closed wound without AL. Figure 4c indicates the light propagation of the green laser and the converted light from ZnS:Ag,Co particles after UV light irradiation. The emission intensity of ZnS:Ag,Co particles at 540 nm remained strong regardless of the skin depth, whereas the intensity of the green laser at 540 nm decreased exponentially along the skin depth of the closed incision. The AL intensity of ZnS:Ag,Co particles was much stronger than that of the green laser at 30 s. These results demonstrated the green light of ZnS:Ag,Co particles could be delivered effectively into the deep tissue layer for long-term tissue bonding. In addition, the penetration depth of ZnS:Ag,Co particles and HA-RB conjugates added to the same incision site of porcine skin was assessed by two-photon microscopy (Supporting Information, Fig. S6). The HA-RB conjugates in HA-RB/ZnS:Ag,Co mixtures penetrated deeper than RB from the incisional plane. HA appeared to enhance the transdermal penetration of HA-RB conjugates^10,25^, which could activate more collagen layers for enhanced PTB upon light irradiation.

### Collagen fibrillogenesis by HA-RB/ZnS:Ag,Co mixtures with UV light irradiation

**Fig. 5:**
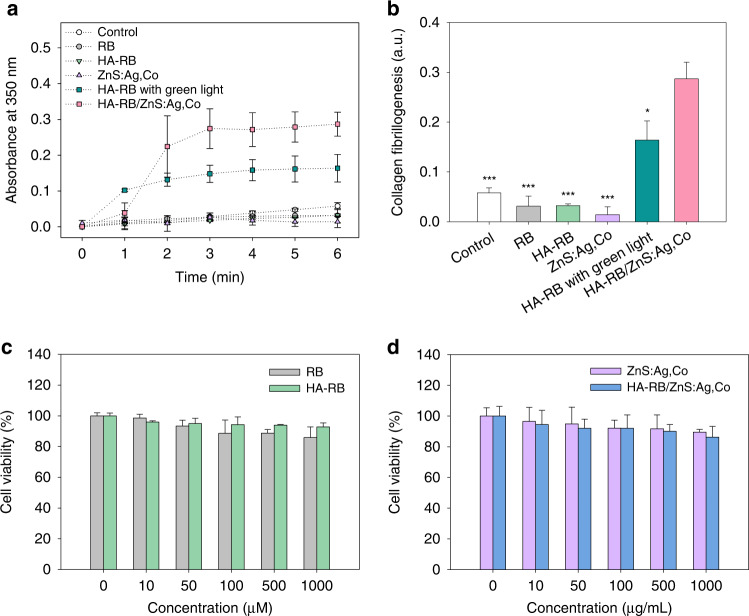
Collagen fibrillogenesis and biocompatibility of HA-RB/ZnS:Ag,Co mixtures. **a** The degree of collagen crosslinking by monitoring the optical density at 350 nm after treatment with the control (DI water), RB, HA-RB conjugates, ZnS:Ag,Co particles, or HA-RB/ZnS:Ag,Co mixtures for 6 min under on-off fractional irradiation of UV light. Experimental conditions were the same for the case of HA-RB conjugates with green light. **b** The relative increase of optical density at 6 min after treatment with samples in **a** (*n* = 3, *P < 0.05, ***P < 0.001 versus HA-RB/ZnS:Ag,Co mixture). The relative viability of NIH3T3 cells treated with **c** RB or HA-RB conjugates, and **d** ZnS:Ag,Co particles or HA-RB/ZnS:Ag,Co mixtures by CCK-8 assay (*n* = 3). Data are expressed as mean ± SD

The efficiency of collagen fibrillogenesis was analyzed by measuring the optical density of collagen solutions (Fig. 5a). The turbidity and optical density increased with collagen crosslinking. All samples were dissolved in deionized (DI) water and irradiated with UV light or green light that turned on and off every 30 s (on/off cycle: 30 s/30 s). The turbidity of HA-RB/ZnS:Ag,Co mixtures increased rapidly compared to the control (DI water), RB, HA-RB conjugates, and ZnS:Ag,Co particles under on-off fractional irradiation of UV light. The significant implication was that the collagen fibrillogenesis rate of HA-RB/ZnS:Ag,Co mixtures with on-off fractional UV irradiation was much faster than that of HA-RB conjugates with green light. More activation of RB was observed in HA-RB/ZnS:Ag,Co mixtures with on-off fractional irradiation of UV light, as the power density, including PL and AL was slightly higher than that of the green laser (Supporting Information, Table S1). The HA-RB/ZnS:Ag,Co mixtures with on-off fractional irradiation of UV light enhanced the efficiency of photo-crosslinking by both oxygen-dependent energy transfer and oxygen-independent electron transfer pathways^8^. The RB triplet state induced collagen free radicals for covalent crosslinking via the oxygen-independent electron transfer pathway with oxygen depletion under light irradiation at PL power density of HA-RB/ZnS:Ag,Co mixtures. In contrast, the AL of HA-RB/ZnS:Ag,Co mixtures showed relatively low power density, resulting in increased singlet oxygen generation and additional crosslinking between carbonyl groups of collagens via the oxygen-dependent energy transfer pathway^26^. Figure 5b shows the relative increase in the degree of collagen fibrillogenesis at 6 min, the stabilization time point in Fig. 5a. All these results confirmed that HA-RB/ZnS:Ag,Co mixtures with on-off fractional UV irradiation could accelerate the collagen crosslinking in a more efficient way than the conventional PTB using green light.

### Biocompatibility of ZnS:Ag,Co particles

The cytotoxicity of RB, HA-RB conjugates, ZnS:Ag,Co particles, and HA-RB/ZnS:Ag,Co mixtures was assessed by cell counting kit-8 (CCK-8) assay (Fig. 5c, d). As shown in Fig. 5c, the viability of NIH3T3 cells treated with HA-RB conjugates was higher than that treated with RB, which revealed that HA alleviated the cytotoxicity of RB. However, the relative cell viability of HA-RB/ZnS:Ag,Co mixtures was slightly lower than that of ZnS:Ag,Co particles (Fig. 5d). Since HA-RB conjugates and ZnS:Ag,Co particles were mixed in the same amount without binding to each other, the total amount increased and the cell viability appeared to decrease. Nevertheless, ZnS:Ag,Co particles and HA-RB/ZnS:Ag,Co mixtures showed significant biocompatibility with cell viability of 85% at the ZnS:Ag,Co concentration of 1000 μg mL^−1^.

### In vivo PTB by HA-RB/ZnS:Ag,Co mixtures with UV light irradiation

**Fig. 6:**
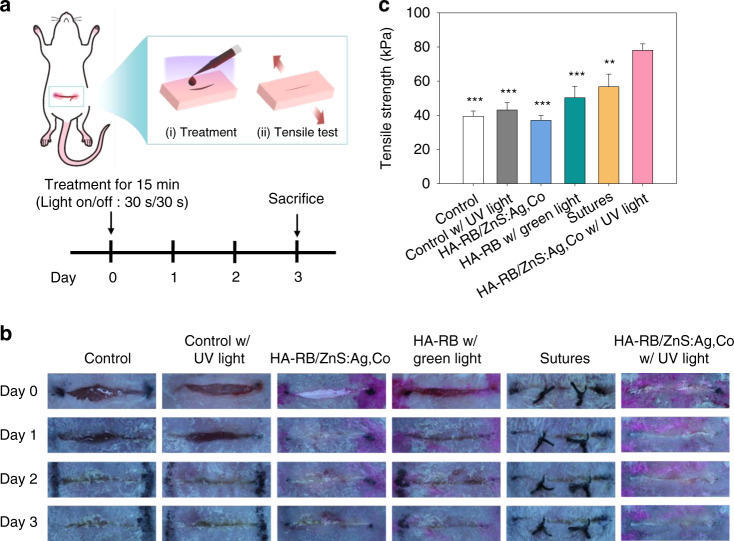
In vivo wound healing and tensile strength. **a** Schematic illustration for in vivo PTB procedure. **b** Photographs of wound regions after treatment with the control (PBS), PBS with UV light, HA-RB/ZnS:Ag,Co mixtures, HA-RB conjugates with green light, sutures, or HA-RB/ZnS:Ag,Co mixtures with UV light irradiation (on/off cycle: 30 s/30 s). **c** In vivo tensile strength of wound skins treated with each group at day 3 (*n* = 4, *P < 0.05, **P < 0.01, ***P < 0.001 versus HA-RB/ZnS:Ag,Co mixture with UV light)

We assessed the feasibility of HA-RB/ZnS:Ag,Co mixtures for PTB in vivo. An incision (1cm) was made on the abdominal skin of mice, which was treated with (i) the control (phosphate-buffered saline, PBS), (ii) PBS with UV light, (iii) HA-RB/ZnS:Ag,Co mixtures, (iv) HA-RB conjugates with green light, (v) sutures, or (vi) HA-RB/ZnS:Ag,Co mixtures with UV light irradiation (on/off cycle: 30 s/30 s). The wound was slowly closed while applying on-off fractional light irradiation to adhere to the incised surface. After treatment, tissue bonding was monitored from day 0 to day 3, as shown in Fig. 6a. The incised skin of each group was visualized and compared at different time points (Fig. 6b). The group treated with HA-RB/ZnS:Ag,Co mixtures under on-off fractional UV irradiation resulted in the best tissue bonding effect, followed by HA-RB conjugates with green light and PBS with UV light. The unclosed wounds and crusts were still shown in control, the control with UV light, and HA-RB/ZnS:Ag,Co mixtures. The wound of the sutured group was well-closed, but traces of the surgical thread remained clear.

The tensile strength of incised skins in each group was measured to analyze the PTB effect statistically (Fig. 6c). The tensile strengths of the control and the control with UV light were 39.46 ± 3.10 and 43.11 ± 4.30 kPa, respectively, indicating no significant effect of UV light on the PTB. The nonirradiated HA-RB/ZnS:Ag,Co mixtures exhibited a similar tensile strength of 37.04 ± 2.76 kPa. The HA-RB conjugates with green light and sutures showed significant values with the tensile strength of 50.28 ± 6.63 kPa and 56.68 ± 7.41 kPa, but the highest tensile strength of 78.05 ± 3.75 kPa was measured in the skin treated with HA-RB/ZnS:Ag,Co mixtures under on-off fractional UV irradiation. All these results confirmed the feasibility of controlled ALPs for photo-activated tissue bonding.

## Discussion

ALPs have been widely investigated for bioimaging^27^ and phototherapy^28^ owing to their long-term maintenance of light without the need for continuous external light irradiation. To improve the intrinsic optical properties, recent studies have focused on increasing decay time or AL intensity. The reported decay time of ALPs varies from a few minutes^29^ to several days^30^, and the power density of AL demonstrated by Hu et al.^31^ and Yang et al.^32^ were 1.81 × 10^−4^ and 14.9 × 10^−4^ mW cm^−2^, respectively. Since the generation of reactive oxygen species (ROS) can be induced at low light intensity^26^, ALPs demonstrated their great potential as a PDT agent with AL^33,34^. However, there has been no report on using ALPs in the field of PTB. PTB requires a relatively higher intensity of light than PDT^35^, and the known minimum power density of 1.5 mW cm^−2^ used for PTB did not completely heal the wound even after prolonged and repeated treatments^10^.

The conventional PTB using green light has low skin permeability and cannot transmit light after closing the incision^36^. To overcome the limitation of PTB only for treating superficial wounds, upconversion materials (UCMs) have been investigated for the noninvasive delivery of visible light to deep tissue. UCMs convert the skin-penetrating NIR light into visible light for PTB^10,37^. However, due to low conversion efficiency^11,12^, phototherapy with UCMs requires prolonged exposure of the wound to high-intensity NIR light irradiation^38,39^. In addition, the 1/e penetration depth of NIR light is only 3 mm, which can be a remarkable limitation for PTB in the deep tissue^40^. ALPs with high power densities of PL and AL can address the low conversion efficiency of UCMs and the limited penetration depth of light into the skin. The strong PL intensity of ALPs during excitation enables efficient tissue bonding prior to wound closure. Although the light cannot be delivered into the closed wound site, ALPs with strong AL enable the additional collagen crosslinking to improve tissue bonding. In addition, with an afterglow power density high enough for PTB, ALPs can deliver light anywhere, regardless of the skin depth. Thus, ALPs with high power densities of PL and AL can be effectively harnessed for PTB.

We have demonstrated for the first time that ALPs could be applied to PTB and possibly to other biophotonic applications. The ALPs of ZnS:Ag,Co were synthesized with PL and AL power densities sufficient enough for PTB. The ZnS:Ag,Co particles successfully transmitted high-energy light into the deep tissue without adverse effects and were repeatedly recharged after short exposure to the light source. The AL of ZnS:Ag,Co particles continuously activated RB in HA-RB conjugates for enhanced tissue bonding under on-off fractional UV irradiation, reducing the number of treatments and exposure time to the external light source. Despite their advantages, UV light has limited tissue penetration to recharge HA-RB/ZnS:Ag,Co mixtures after the wound is closed completely. Further research will focus on the design of ALPs with superior luminescence to enable tissue bonding in one luminescence treatment regardless of skin penetration depth. In addition, the PTB in the deep tissue would be repeatedly performed by using ALPs that can be recharged by a highly penetrating excitation light source.

## Conclusions

We successfully developed HA-RB/ZnS:Ag,Co mixtures for effective PTB in the deep tissue. The HA-RB conjugates could be delivered into a broad and deep collagen layer at the incision site. The ALPs of ZnS:Ag,Co exhibited strong luminescence intensity and rapid rechargeability, which continuously activated RB in HA-RB conjugates and induced additional collagen crosslinking even after removing the light source. The light propagation assessment revealed that the AL of ZnS:Ag,Co particles was maintained on the closed wound skin. After confirming in vitro collagen fibrillogenesis and biocompatibility, in vivo tissue bonding tests validated the photochemically accelerated tissue bonding effect of HA-RB/ZnS:Ag,Co mixtures under on-off fractional UV irradiation. In addition, HA-RB/ZnS:Ag,Co mixtures with on-off fractionated irradiation resulted in an enhanced tensile strength without inflammation and scar formation compared to the conventional PTB with green light irradiation or suturing. Taken together, we could confirm the feasibility of HA-RB/ZnS:Ag,Co mixtures for wound healing and further deep tissue phototherapy.

## Materials and methods

### Materials

Zinc sulfide (ZnS), cobalt (II) acetate, silver nitrate (AgNO_3_), collagen from rat tail, diaminohexane (DAH), N-hydroxysuccinimide sodium salt (NHS), and 2-[4-(2-hydroxyethyl)piperazin-1-yl]ethanesulfonic acid (HEPES) buffer solution were purchased from Sigma–Aldrich (St. Louis, MO). RB dye and 1-ethyl-3-(3-(dimethylamino)propyl)-carbodiimide hydrochloride (EDC) were acquired from Junsei Chemical Co. (Tokyo, Japan) and Tokyo Chemical Industry (Tokyo, Japan), respectively. HA (MW 100 kDa) was purchased from Lifecore Biomedical (Chaska, MN). PBS (pH 7.4) was purchased from Tech & Innovation (Seoul, Korea). Dulbecco’s modified Eagle’s medium (DMEM), fetal bovine serum (FBS), and antibiotics were purchased from Gibco (Grand Island, NY). Cell counting kit-8 (CCK-8) was obtained from DoGenBio Co. (Seoul, Korea). A mouse embryonic fibroblast of the NIH3T3 cell line was acquired from Korean Cell Line Bank (Seoul, Korea).

### Synthesis of ZnS:Ag,Co particles

The ZnS:Ag,Co particles were prepared by a solid-state reaction in an argon atmosphere. In brief, ZnS powders (92 mg) were dispersed in DI water (0.5 mL) and stirred for 6 h under an ambient atmosphere. Well-dispersed ZnS solution was homogeneously mixed with an optimized amount of AgNO_3_ (23 mM in DI water, 30 μL) and cobalt (II) acetate (8 mM in DI water, 7.5 μL). After centrifuging the mixed solution for 15 min at 8000 rpm, the precipitate was transferred to a crucible and dried at 100 °C. Then, the mixture was sintered at optimized temperature and reaction time in an argon atmosphere (950 °C, 3.5 h), details are found in S1 and S2, Supporting Information.

### Synthesis of HA-RB conjugates

The HA-RB conjugates were prepared by the reaction between HA-DAH and RB using EDC chemistry. First, HA (100 kDa, 100 mg) and DAH (578 mg) were dissolved in DI water (20 mL). The solution was mixed with EDC (191 mg, 4 molar ratios to HA) and NHS (54 mg, 1 molar ratio to HA) at pH 4.8 for 24 h to obtain HA-DAH with a DAH content of 50 mol%. RB (242.8 mg, 2 molar ratios to DAH) was dissolved into the HA-DAH solution and mixed with EDC and NHS (8 molar ratios to DAH) at pH 4.8. After stirring in the dark condition for 24 h, the HA-RB conjugate was washed using a dialysis tube (MWCO = 7000 Da) and lyophilized for 2 days.

### Characterization of ZnS:Ag,Co particles and HA-RB conjugates

The phase transition of ZnS:Ag,Co particles was analyzed by XRD (D/MAX-2500-PC, Rigaku Co., Akishima, Japan) with Cu-Kα radiation. The morphology and the composition of ZnS:Ag,Co particles were characterized by field-emission scanning electron microscopy (FE-SEM) (JSM-7800F, JEOL Ltd., Akishima, Japan) and HRTEM equipped with EELS (JEM-2200FS, JEOL Co., Akishima, Japan). The binding energy spectrum was investigated using XPS (ESCALAB 250, Thermo Scientific, Waltham, MA). The absorbance spectrum of HA-RB conjugates was measured by UV/vis spectrophotometry (S-3100, Scinco Co., Seoul, Korea).

### PL spectroscopy of ZnS:Ag,Co particles

The PL spectrum of ZnS:Ag,Co particles was analyzed by a spectrofluorometer (JASCO, FP-8300, Tokyo, Japan) in the form of a cylindrical phantom (16 mm diameter × 2 mm thickness). The phantom was made by mixing Zns:Ag,Co particles and polydimethylsiloxane (PDMS) with a particle concentration of 75 mg mL^−1^. The emission spectral range was 400–650 nm with excitation at 365 nm.

### AL property of ZnS:Ag,Co particles

The AL spectrum of ZnS:Ag,Co particles was acquired by a spectrofluorometer (JASCO, FP-8300, Tokyo, Japan) in the form of the particle-containing PDMS phantom. After irradiation of 365 nm UV light, the excitation light source was switched off, and the AL was measured at the wavelength range of 400–650 nm. The time-resolved afterglow was obtained using a microplate fluorometer (Fluoroskan Ascent FL, Thermo Scientific, Waltham, MA). ZnS:Ag,Co particles were transferred into the 96-well microplate, and the AL intensity was acquired for 30 min after UV light pre-irradiation for 30 s. ZnS:Ag,Co particles were recharged with UV light for 5 s and the afterglow decay curve was monitored every 5min. The afterglow image of ZnS:Ag,Co particles was taken by a CCD camera (Canon EOS 100D, Canon, Tokyo, Japan) after the irradiation of a hand-held UV lamp (6 W). The power density of AL was evaluated using a power meter (PM100D, Thorlabs, Newton, New Jersey) equipped with a power sensor (S120VC, Thorlabs, Newton, New Jersey). After UV light irradiation for 30 s, power measurement was conducted at 540 nm wavelength.

### Light intensity assessment into the incision of porcine tissue

Each 50 μL of PBS and ZnS:Ag,Co particles (8 mg mL^−1^, green light converted from UV light = 40 mW cm^−2^) was loaded into the 1 cm incision of 1.5 × 1.5 cm^2^ porcine skin. A green laser (532 nm, 40 mW cm^−2^) or UV lamp was illuminated at the incision site. Front cross-sectional images were taken with a CCD camera before and after the lights were turned off. ImageJ software was used to measure the green fluorescence and afterglow profiles of the images at pre-determined depth points of the closed incision.

### Collagen fibrillogenesis test

Collagen fibrillogenesis was monitored at 37 °C by a temperature-controlled microplate spectrophotometer (EMax End point ELISA microplate reader, Molecular Devices, Sunnyvale, CA). The collagen (type 1) from the rat tail was dispersed in acetic acid (20 mM) at a concentration of 3 mg mL^−1^ and diluted in HEPES (0.2 M) and 1.0 M PBS (pH 7.4) in an 8:1:1 volume ratio. The collagen solution was neutralized on ice and transferred into the 96-well microplate. Each 50 μL of the control (DI water), RB (500 μM), HA-RB conjugates (500 μM), ZnS:Ag,Co particles, and HA-RB/ZnS:Ag,Co mixtures was mixed with 100 μL of the collagen solution (*n* = 3). The absorbance from 350 to 370 nm was acquired at 1 min intervals for 6 min under UV light. During irradiation, the light was turned on and off every 30 s (On/Off cycle: 30 s/30 s). The group of HA-RB conjugates with green light was monitored separately under the same conditions.

### In vitro biocompatibility test

NIH3T3 cells were cultured in DMEM with 10 vol% FBS and 1 vol % antibiotics. The cells were transferred into 96-well plates at 2.5 × 10^5^ cells well^−1^, and various concentrations of HA-RB conjugates, ZnS:Ag,Co particles, or HA-RB/ZnS:Ag,Co mixtures were added to the wells (*n* = 3). All plates were incubated for 24 h. Then, the medium in each well was substituted with serum-free medium and CCK-8 solution. The relative cell viability was obtained by measuring optical density at 450 nm with a microplate reader.

### In vivo tissue bonding test

The in vivo tissue bonding experiment was conducted following the laboratory animal protocol approved by the institutional animal care and use committee of the Pohang University of Science and Technology in accordance with the National Institutes of Health Guide for the Care and Use of Experimental Animals. Female BALB/c mice (6 week old) were anesthetized and abdominal skin was shaved to make a deep tissue incision (1 cm). The incised skin was treated with 50 μL PBS as the control, PBS with UV light, ZnS:Ag,Co/HA-RB mixtures, HA-RB conjugates with green light, sutures, or HA-RB/ZnS:Ag,Co mixtures with UV light for 15 min (on/off cycle: 30 s/30 s). After treatment on day 0, treated skins were monitored for 3 days, and mice were sacrificed by CO_2_ euthanasia to collect 1 × 2 cm^2^ treated tissue specimens (*n* = 4). After storage in a humid atmosphere for 1h, the tensile strength of adhered specimens was measured by Instron (Instron 3340, Instron Co., Norwood, MA, USA) with a 10 N load cell at a constant rate of 5 mm min^−1^. Both ends of the specimen were fixed with forceps and pulled in both directions at the same time. The maximum tensile strength was recorded by measuring the tensile strength until the adhered tissue was completely separated^41^.

### Statistical analysis

One-tail statistical analysis was performed using Student’s *t*-tests. Data were expressed as mean ± standard deviation (SD). The values for *P < 0.05; **P < 0.01; and ***P < 0.001 expressed statistical significance. Statistical analysis was performed by the software SigmaPlot 10.0 (Systat Software Inc., CA).

## Supplementary information

Supporting Information

## Data Availability

All data needed to evaluate the conclusions in the paper are present in the paper. Additional data related to this paper may be requested from the authors.
