# Supplementary material for: Controlled afterglow luminescent particles for photochemical tissue bonding
Source: Light Sci Appl. 2022 Oct 27;11:314. doi: 10.1038/s41377-022-01011-3 (PMC9613626; doi:10.1038/s41377-022-01011-3)
Supplement: Supplementary file 1 — Supporting Information [file 41377_2022_1011_MOESM1_ESM.docx]

Supporting Information

**Controlled Afterglow Luminescent Particles**

**for Photochemical Tissue Bonding**

Seong-Jong Kim^1,†^, Minji Choi^1,†^, Guosong Hong^2^, and Sei Kwang Hahn^1,*^

^1^ Department of Materials Science and Engineering, Pohang University of Science and Technology (POSTECH), 77 Cheongam-ro, Nam-gu, Pohang, Gyeongbuk 37673, Korea

^2^ Department of Materials Science and Engineering, Stanford University, Stanford, CA 94305, USA

†These authors (Seong-Jong Kim and Minji Choi) contributed equally.

*** CORRESPONDING AUTHOR FOOTNOTE**

Tel.: +82 54 279 2159; E-mail: skhanb@postech.ac.kr (S.K. Hahn).

**S1: The optimized calcination conditions for the synthesis of ZnS:Ag,Co particles**

As afterglow luminescence (AL) depends on calcination and dopant concentration, the study was systemically conducted to derive the optimized condition. In the case of the sample sintered up to 800 ℃, the cubic phase was dominant and there was almost no peak in the wurtzite phase (Fig. S1a). Photoluminescence (PL) properties before the phase transition are mainly related to the change in the ZnS band gap due to the increase in crystal size^1^. The AL intensity increased with the annealing temperature and had a maximum value at an annealing temperature of 950 ℃, which probably contributed to the incorporation of Co^2+^ and Ag^+^ dopants in the host ZnS^2^ (Fig. S1b, c). However, further increasing the annealing temperature to 1100 ℃ lowered the intensity of AL due to improved crystallinity that reduced surface defects and traps^3^. The crystal structure of ZnS:Ag,Co particles annealed at 950 ℃ was wurtzite phase independent of the annealing time (Fig. S1d). As shown in Fig. S1e and f, the intensity of green AL increased with calcination time due to more vacancies in Zn^2+^ creation^4^ and remained nearly unchanged at annealing times of 3h 30 min or more. Based on the results presented above, we determined annealing for 3h 30 min at 950 ℃ as optimized parameters for the synthesis of ZnS:Ag,Co particles.


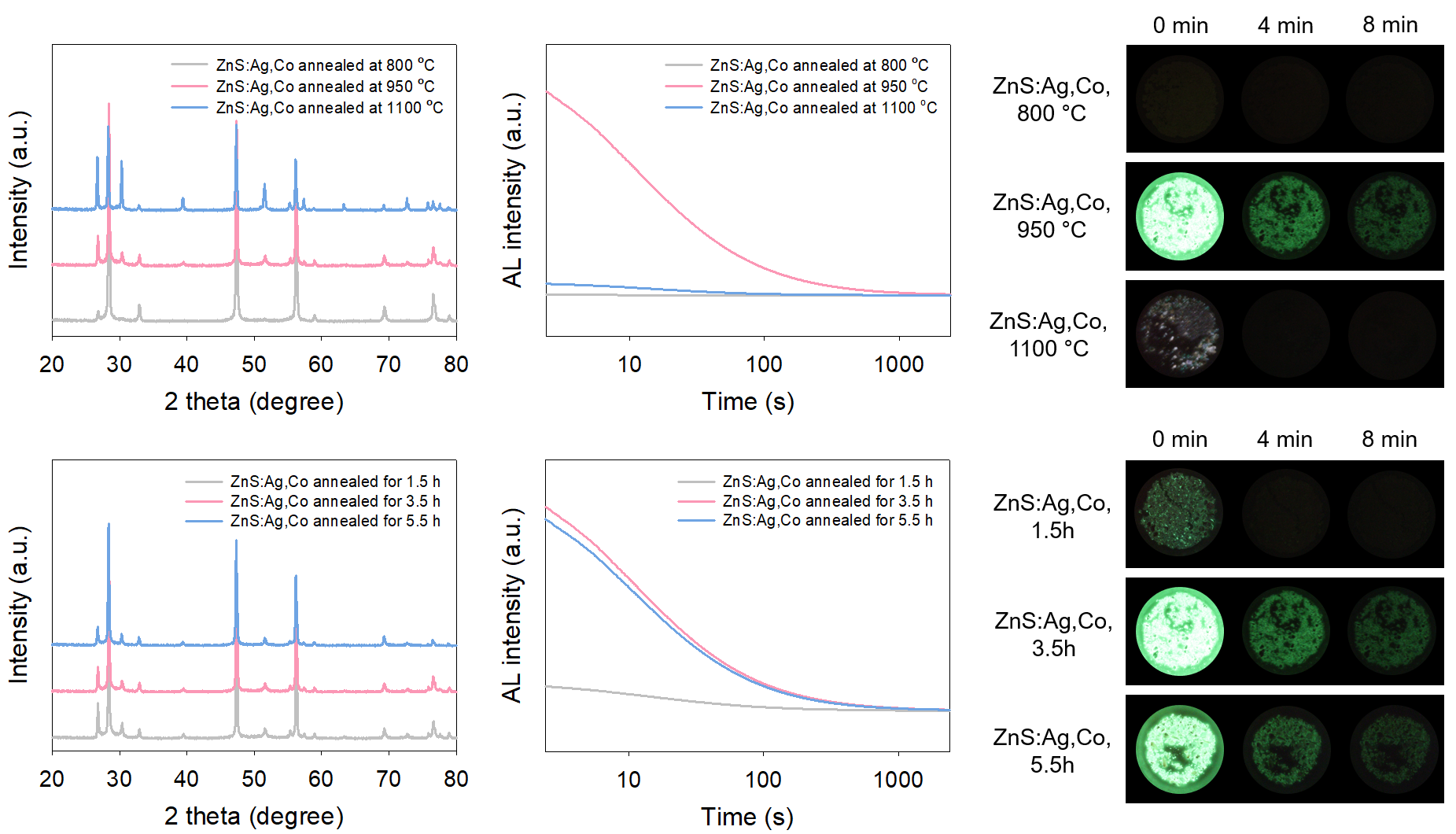


**f**

**e**

**d**

**a**

**b**

**c**

Fig. S1. Characterization of ZnS:Ag,Co particles at different calcination conditions. (a) XRD pattern, (b) afterglow decay curves, and (c) afterglow images of ZnS:Ag,Co particles prepared at different annealing temperatures. Afterglow images were taken by CCD camera after irradiation of 365 nm UV lamp for 30 s. (d) XRD pattern, (e) afterglow decay curves, and (f) afterglow images of ZnS:Ag,Co particles prepared at different annealing times.

**S2: The optimized Ag^+^ and Co^2+^ doping concentration for the synthesis of ZnS:Ag,Co particles**

The PL spectrum and afterglow decay curves varied with doping concentrations of Ag^+^ and Co^2+^ dopants. The intensity of main emission peaks at 450 nm and 540 nm changed according to Ag^+^ ions (Fig. S2a). The emission increased at 540 nm and decreased at 450 nm as the Ag^+^ concentration increased due to the insertion of silver into the lattice of ZnS and the excess of sulfur vacancies. However, the emission intensity at 540 nm decreased as the Ag^+^ content exceeded a certain amount. It is due to the interaction between adjacent silver ions^5,6^. The Afterglow decay curve showed a similar trend to the emission intensity at 540 nm (Fig. S2b). The emission at 540 nm and AL intensity increased with increasing amount of Co^2+^ ions due to the introduction of shallow electronic states (Fig. S2c, d). When the amount of Co^2+^ ions exceeded a certain concentration, the emission intensity decreased, which is related to the non-radiative transition between the inter-band states of Co^2+^ ions^7^. The maximum intensity of luminescence was found when the amounts of Ag^+^ and Co^2+^ dopants were 4.87 × 10^-4^ mol% and 0.636 × 10^-4^ mol%, respectively.


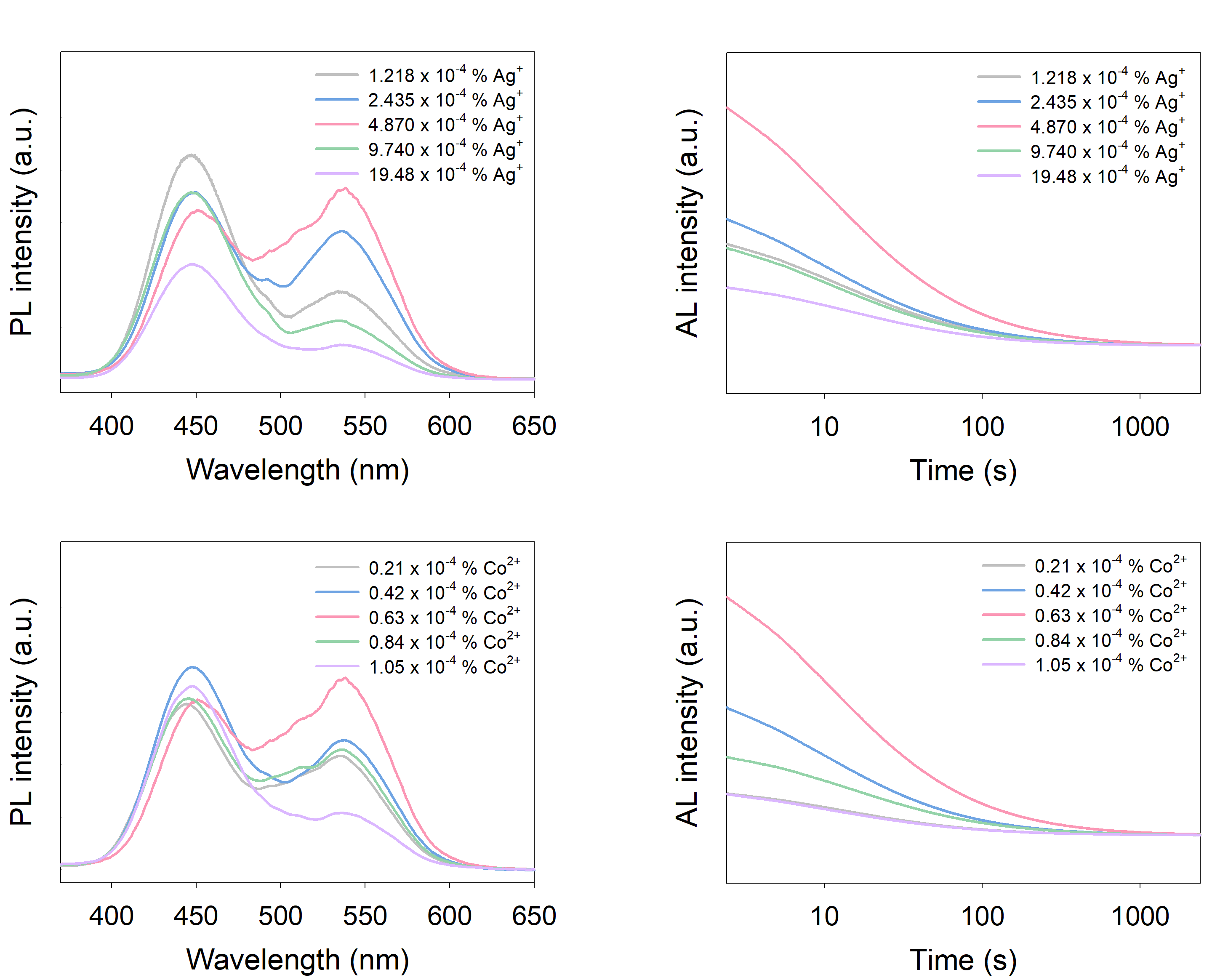


**d**

**b**

**c**

**a**

Fig. S2. Luminescence properties of ZnS:Ag,Co particles with different concentrations of Ag^+^ and Co^2+^ dopants. (a) PL spectrum and (b) afterglow decay curves of Zn_1_S:Ag_x_Co_y_ (y=6.3 × 10^-5^) particles with various contents of Ag^+^ dopant. (c) PL spectrum and (d) afterglow decay curves of Zn_1_S:Ag_x_Co_y_ (x=4.87 × 10^-4^) particles with various contents of Co^2+^ dopant.

**S3: The XPS spectra of ZnS:Ag,Co particles**


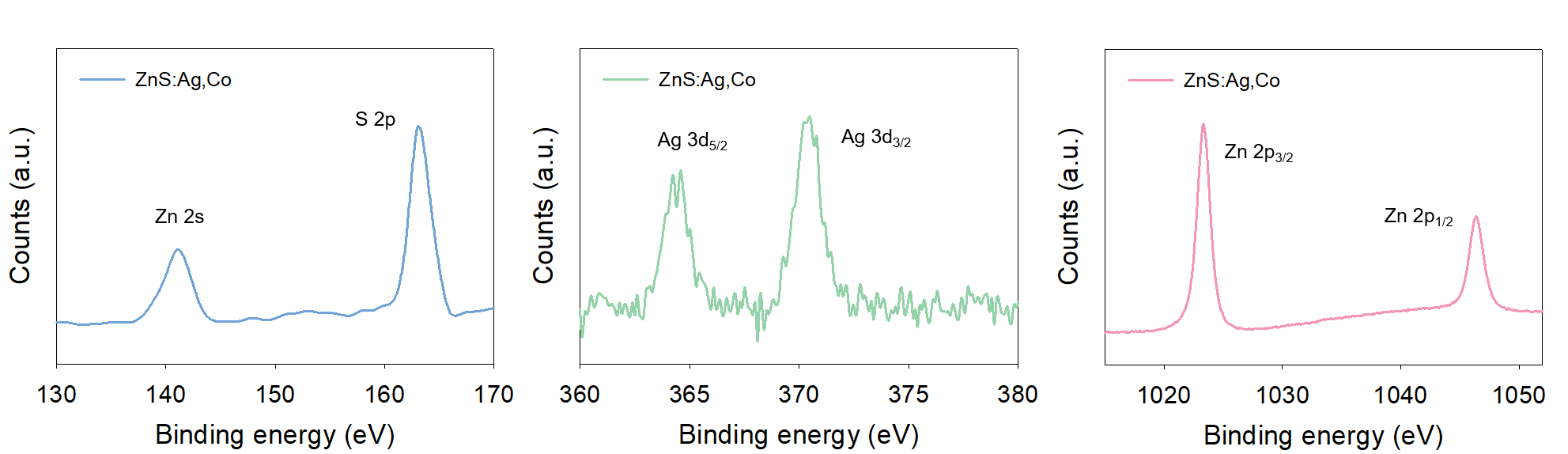


Fig. S3. The XPS spectrum of zinc 2*s*, sulfur 2*p*, silver 3*d* and zinc 2*p* in ZnS:Ag,Co particles.

**S4: Characterization of HA-RB conjugates**


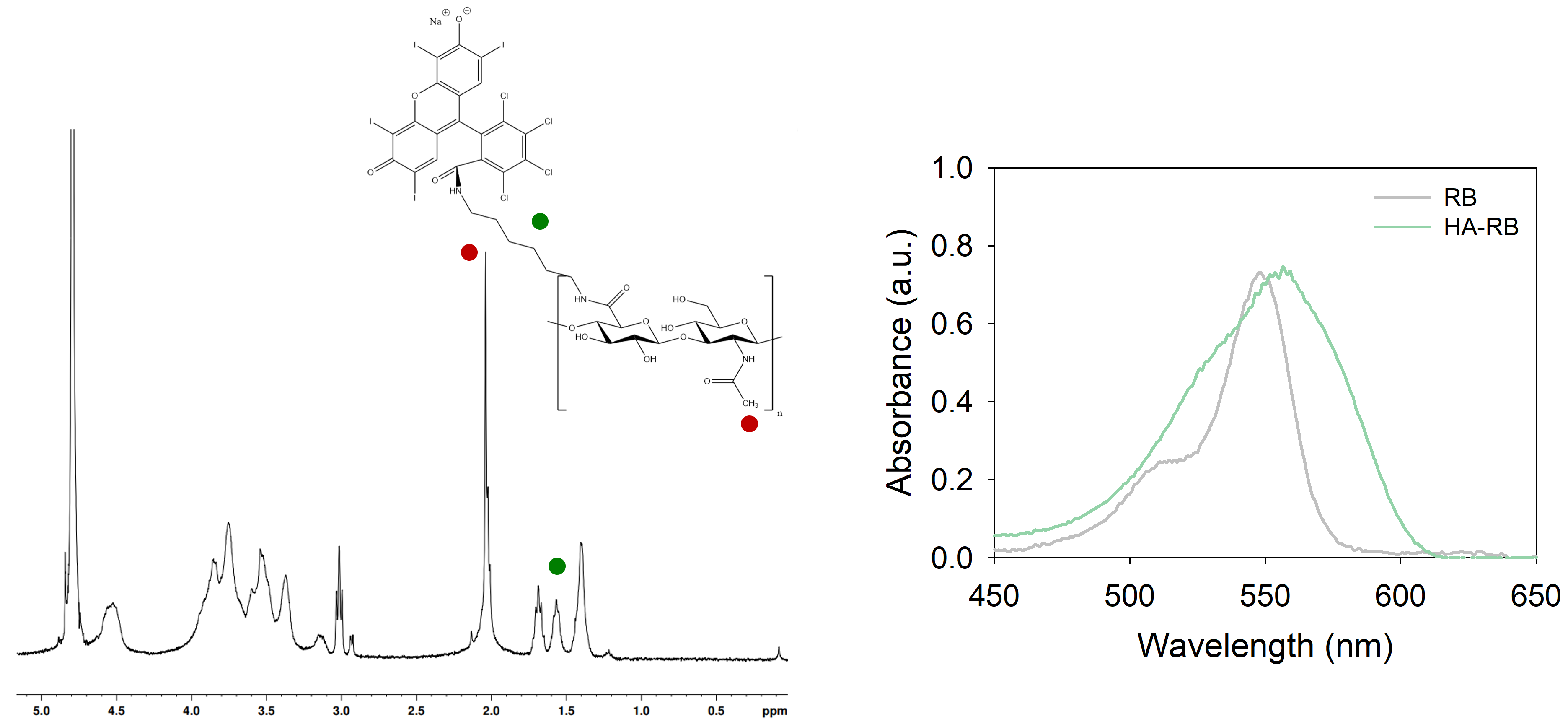


**b**

**a**

Fig. S4. Characterization of HA-RB conjugates. (a) ^1^H NMR spectrum of HA-RB conjugates. (b) Absorbance spectrum of RB and HA-RB conjugates.


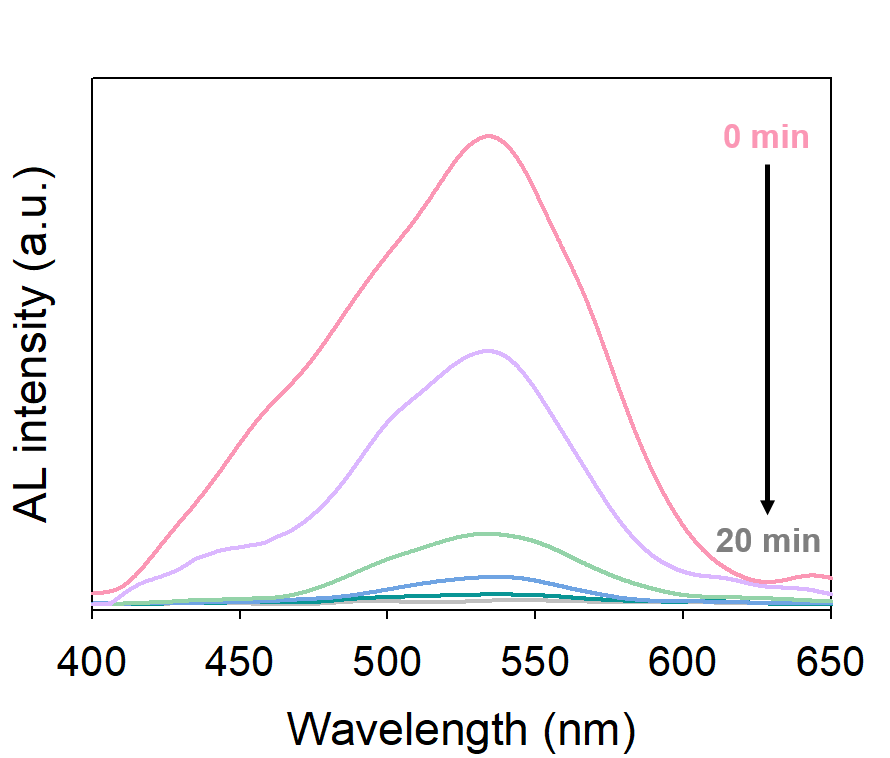
**S5: The Time-resolved AL spectrum of ZnS:Ag,Co particles**

Fig. S5. The Time-resolved AL spectrum of the PDMS phantom comprising ZnS:Ag,Co particles.

**S6: The penetration depth of HA-RB conjugates in HA-RB/ZnS:Ag,Co mixtures**


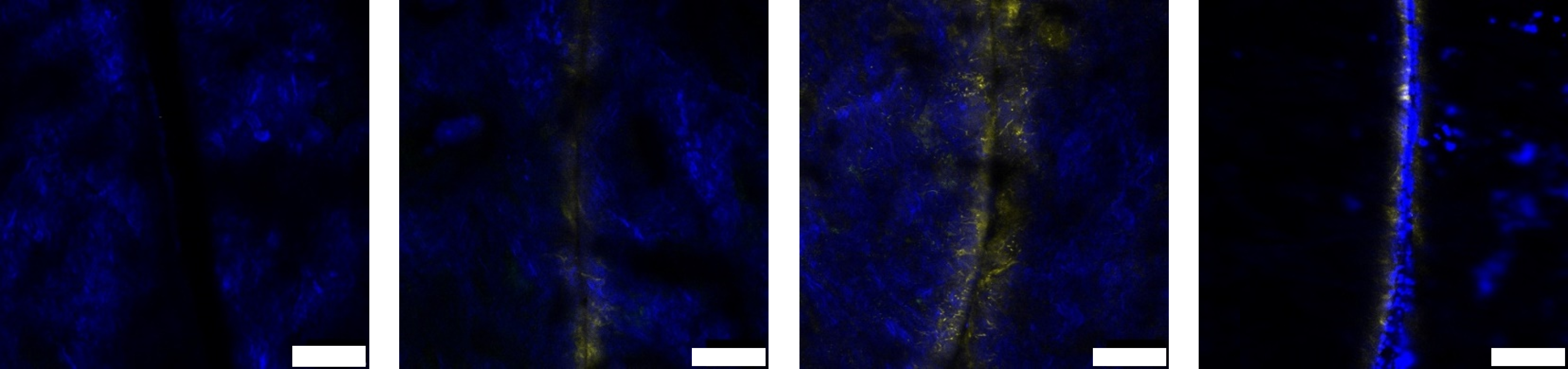


HA-RB/ZnS:Ag,Co

HA-RB

RB

PBS

Fig. S6. *Ex vivo* two-photon microscopic images after treatment of samples into the porcine skin (blue = collagen, yellow = RB, cobalt blue = ZnS:Ag,Co, scale bar = 100 μm).

**Table S1.** Calculated power density and energy density of green laser and ZnS:Ag,Co particles at 540 nm.

|  | Green laser | ZnS:Ag,Co | ZnS:Ag,Co (w/ AL) |
| --- | --- | --- | --- |
| Power density  at 540 nm  (mW cm^-2^) | 40 | (PL) 40 | (AL) 1.5 |
| Energy density  at 540 nm  (30 s, mJ cm^-2^) | 1200 | (PL) 1200 | (PL+AL) 1233.8 |

Supplementary information accompanies the manuscript on the *Light: Science & Applications* website (<http://www.nature.com/lsa>).

**References:**

1. Nguyen, T. T., Trinh, X. A., Nguyen, L. H. & Pham, T. H. Photoluminescence characteristics of as-synthesized and annealed ZnS: Cu, Al nanocrystals. *Adv. Nat. Sci.: Nanosci. Nanotechnol.* **2**, 035008 (2011).

2. Sharma, M. *et al*. Tunable blue-green emission from ZnS (Ag) nanostructures grown by hydrothermal synthesis. *J. Mater. Res*. **33**, 3963-3970 (2018).

3. Dai, Z. *et al*. Effect of annealing temperature on persistent luminescence of Y_3_Al_2_Ga_3_O_12_: Cr^3+^ co-doped with Ce^3+^ and Pr^3+^. *Opt. Mater*. **111**, 110522 (2021).

4. Abdukayum, A., Chen, J. T., Zhao, Q. & Yan, X. P. Functional near infrared-emitting Cr^3+^/Pr^3+^ co-doped zinc gallogermanate persistent luminescent nanoparticles with superlong afterglow for in vivo targeted bioimaging. *J. Am. Chem. Soc*. **135**, 14125-14133 (2013).

5. Sethi, R., Kumar, L., Sharma, P. K. & Pandey, A. C. Tunable visible emission of Ag-doped CdZnS alloy quantum dots. *Nanoscale Res. Lett*. **5**, 96-102 (2010).

6. Kar, S., Biswas, S., Chaudhuri, S. & Nambissan, P. M. G. Substitution-induced structural transformation in Mn-doped ZnS nanorods studied by positron annihilation spectroscopy. *Nanotechnology* **18**, 225606 (2007).

7. Saavedra-Rodriguez, G., Pal, U., Sánchez-Zeferino, R. & Álvarez-Ramos, M. E. Tunable white-light emission of Co^2+^ and Mn^2+^ co-doped ZnS nanoparticles by energy transfer between dopant ions. *J. Phys. Chem*. C **124**, 3857-3866 (2020).
